# Caring for a Family Member With Mild Dementia: Perceptions, Connections, and Relational Dynamics With the Sacred

**DOI:** 10.1093/geroni/igab046.1787

**Published:** 2021-12-17

**Authors:** Jocelyn McGee, Dennis Myers, Rebecca Meraz, Davie Morgan

**Affiliations:** 1 Baylor University, United States; 2 Baylor University, Waco, Texas, United States; 3 Baylor University, Dallas, Texas, United States

## Abstract

Researchers define spirituality as the search for or connection with the “sacred”, which is transcendent and considered blessed, holy, or revered. For some, the sacred is connection with a divinity (e.g., God, gods) and for others, a close relationship with something else bigger than themselves (e.g., the Universe, Nature, a life philosophy). Current research reports that family caregivers with a strong connection to the sacred, as compared with those who do not, have fewer symptoms of depression, more positive perceptions of the caregiving experience, improved coping, and bolstered resilience. However, there is limited research on the impact of spirituality on the perceptions of familial caregivers whose loved ones have recently been diagnosed with dementia. In this study, 27 family caregivers of persons with mild dementia (CDR=1) were interviewed using the Dimensions of Caregiving Interview (DCI, McGee & Carlson, 2013). The DCI identified positive psychological aspects of the caregiving experience, including spirituality. Three heuristic themes emerged from Directed Content Analysis: perceptions about the sacred reflect variability in the early part of the caregiving journey; specific characteristics, traits, and functions of the sacred shape caregiver coping and adjustment; and the relational dynamics between caregivers and the sacred inform adaptation. Recommendations for clinical practice and additional research are provided.

